# Human metabolism and excretion kinetics of benzotriazole UV stabilizer UV-327 after single oral administration

**DOI:** 10.1007/s00204-022-03401-3

**Published:** 2022-11-05

**Authors:** Corinna Fischer, Edgar Leibold, Julia Hiller, Thomas Göen

**Affiliations:** 1 Institute and Outpatient Clinic of Occupational, Social and Environmental Medicine, Friedrich-Alexander-Universität Erlangen-Nürnberg, Henkestraße 9–11, 91054 Erlangen, Germany; 2 BASF SE, Product Safety, Carl-Bosch‑Straße 38, 67056 Ludwigshafen Am Rhein, Germany

**Keywords:** Benzotriazole ultraviolet stabilizer, UV-327, Human metabolism, Human biomonitoring, Toxicokinetics

## Abstract

UV-327 (2-(5-chloro-benzotriazol-2-yl)-4,6-di-(*tert*-butyl)phenol) is used as an ultraviolet (UV) absorber in plastic products and coatings. Due to its ubiquitous distribution in the environment, human exposure is conceivable. In the study presented herein, initial information on the human *in vivo* metabolism of UV-327 was obtained by single oral administration to three volunteers. Urine and blood samples were collected up to 72 h after exposure. One study participant additionally donated plasma samples. Maximum blood and plasma levels of UV-327 and its two monohydroxylated metabolites UV-327-6-*m*OH and UV-327-4-*m*OH were reached 6 h post-exposure. Almost the entire amount found in blood and plasma samples was identified as UV-327, whereas the two metabolites each accounted for only 0.04% of the total amount, indicating that UV-327 is well-absorbed from the intestine, but only partially metabolized. Plasma to blood ratios of UV-327, UV-327-6-*m*OH, and UV-327-4-*m*OH ranged from 1.5 to 1.6. Maximum urinary excretion rates of UV-327, UV-327-6-*m*OH, UV-327-4-*m*OH, and UV-327-4 + 6-*di*OH were reached 9–14 h post-exposure. However, only about 0.03% of the orally administered dose of UV-327 was recovered as UV-327 and its metabolites in urine, indicating that biliary excretion may be the major route of elimination of UV-327 and its hydroxylated metabolites. The present study complements the insight in the complex absorption, distribution, metabolism, and elimination (ADME) processes of benzotriazole UV stabilizers (BUVSs).

**Supplementary Information:**

The online version contains supplementary material available at 10.1007/s00204-022-03401-3.

## Introduction

Benzotriazole ultraviolet (UV) stabilizers (BUVSs) are used as additives in consumer and industrial plastic products and coatings to avoid yellowing and degradation as a result of UV radiation (Nakata et al. 2009). The prominent BUVS 2-(5-chloro-benzotriazol-2-yl)-4,6-di-(*tert*-butyl)phenol (UV-327, CAS No. 3864-99-1) is used as a UV absorber in rubber, in car and wood coatings, as well as in plastics, such as polycarbonates and polyvinyl chloride. It was categorized as “very persistent and very bioaccumulative” (vPvB) under the REACH (Registration, Evaluation, Authorization, and Restriction of Chemicals) regulation and was, therefore, categorized as a “substance of very high concern” (SVHC) (ECHA 2015). As such, UV-327 was included in Annex XIV of REACH so that, after a transitional period that lasts until the end of 2023, an authorization is required for placing UV-327 on the market or using it in the European Economic Area (European Commission 2020).

Wastewater treatment plants (WWTPs) are a potentially important indicator of environmental pollution by BUVSs, such as UV-327 (Liu et al. 2012). Accordingly, UV-327 was found in WWTP effluents (Nakata and Shinohara 2010) and sewage sludge (Zhang et al. 2011; Ruan et al. 2012) as well as in various environmental matrices, such as sediment (Apel et al. 2018; Vimalkumar et al. 2018), seawater (Montesdeoca-Esponda et al. 2019; Tashiro and Kameda 2013), and biosolid-amended soils (Lai et al. 2014). UV-327 has furthermore been detected in plastic waste collected from the sea and beaches (Rani et al. 2017; Santana-Viera et al. 2021; Tanaka et al. 2020).

Human exposure to UV-327 and other BUVSs may occur through the use of consumer products and the consumption of fish and seafood (NTP 2011). Dust inhalation represents another potential route of exposure as the compound has been detected in house dust (Carpinteiro et al. 2010; Kim et al. 2012). UV-327 was additionally found in breast-milk samples, which indicates both maternal exposure and possible infant exposure as well (Kim et al. 2019; Lee et al. 2015; Sun et al. 2022).

The oral LD_50_ of UV-327 was determined to be > 5000 mg/kg body weight in rats (Ema et al. 2006), suggesting that UV-327 possesses a low acute toxicity. In contrast to other BUVSs, UV-327 showed no activity against human estrogen and androgen receptors and thus no endocrine-disrupting potential (Fent et al. 2014; Sakuragi et al. 2021). Sakuragi et al. (2021) posited that the chlorine substituent of UV-327 hinders its interaction with the estrogen receptor. The compound furthermore showed no activity against thyroid hormone receptors (Nagayoshi et al. 2015), but was found to bind to human serum albumin by forming hydrogen bonds (Zhuang et al. 2016). UV-327 did not cause reproductive (Ema et al. 2008) nor developmental (Ema et al. 2006) toxicity in rats. However, increased liver weights, histopathological changes in hepatic tissue, and alterations in liver-specific blood parameters were observed after repeated oral administration, especially in male rats (Ema et al. 2008).

**Fig. 1:**
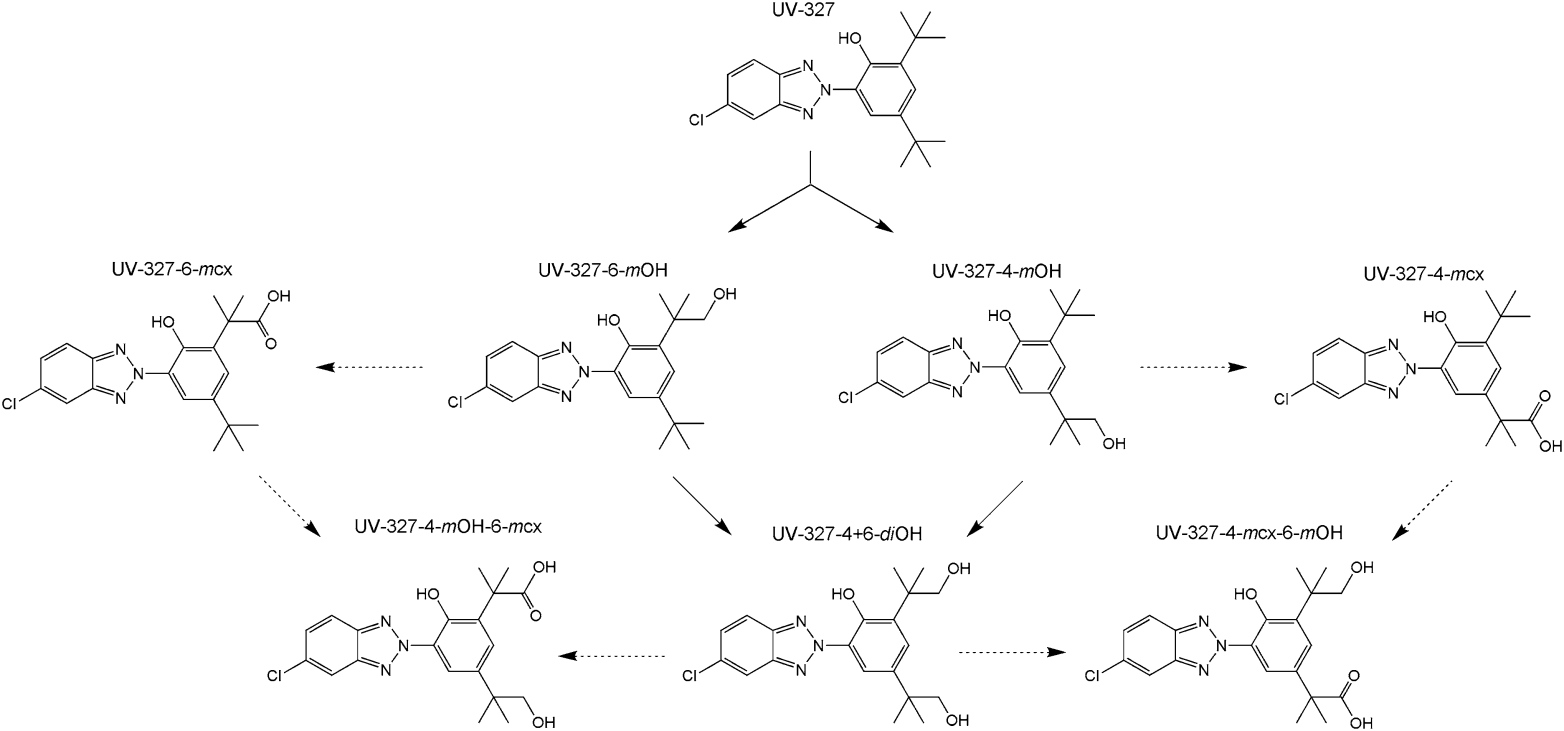
Postulated biotransformation pathway of UV-327. The dashed arrows indicate that these metabolites were detected in vitro but not in vivo

UV-327 was included in the human-biomonitoring initiative agreed upon in 2010 by the German Federal Ministry for the Environment, Nature Conservation and Nuclear Safety (BMU) and the German Chemical Industry Association (VCI), due to potential exposure of the general population and the lack of appropriate human-biomonitoring strategies (Kolossa-Gehring et al. 2017). In this context, phase I metabolites of UV-327 were identified in in vitro experiments with human liver microsomes. The formation of tentatively identified metabolites was confirmed by comparing their retention times and mass-spectrometric fragmentation patterns with those of synthesized reference standards (Fischer et al. 2020). Based on these experiments, the biotransformation pathway is considered a successive oxidation of one or both *tert*-butyl side chains (see Fig. 1). Due to the free hydroxyl and carboxyl groups of UV-327 and its metabolites, conjugation is expected prior to urinary excretion. Following the in vitro experiments, analytical procedures for the determination of UV-327 and its metabolites in urine (Fischer and Göen 2021) and blood (Fischer and Göen 2022) were developed and validated, enabling the analysis of the samples obtained as part of the in vivo study hereby presented. In the present study, volunteers ingested a single oral dosage of UV-327 with the aim to investigate in vivo metabolism and determine the elimination kinetics of UV-327 and its metabolites. This study was needed to confirm the in vivo formation of the metabolites, which have been identified in vitro, as well as reveal their contribution in the human metabolism of UV-327. This study, therefore, tried to clarify whether the metabolites identified in vitro are suitable biomarkers for a human biomonitoring of UV-327.

## Materials and methods

### Chemicals and reagents

UV-327 [certified reference material, TraceCERT^®^, chemical purity 99.3%] was purchased from Merck KGaA (Darmstadt, Germany). 2-(5-Chloro-benzotriazol-2-yl)-4,6-di-(*tert*-1,1,1-[^2^H_9_]-butyl)-3,5-(^2^H_1_-2*H*)phenol [D_20_-UV-327; chemical purity 96%; isotopic purity 93.4%] was ordered from Toronto Research Chemicals (Toronto, Canada). The Institute for Organic and Biomolecular Chemistry (Göttingen, Germany) synthesized 2-(5-chloro-benzotriazol-2-yl)-4-(1-hydroxy-2-methylpropyl)-6-(*tert*-butyl)phenol [UV-327-4-*m*OH; chemical purity > 98%], 2-(5-chloro-benzotriazol-2-yl)-6-(1-hydroxy-2-methylpropyl)-4-(*tert*-butyl)phenol [UV-327-6-*m*OH; chemical purity > 98%], 2-(5-chloro-benzotriazol-2-yl)-4-(1-carboxy-2-methylpropyl)-6-(*tert*-butyl)phenol [UV-327-4-*m*cx; chemical purity > 98%], 2-(5-chloro-benzotriazol-2-yl)-6-(1-carboxy-2-methylpropyl)-4-(*tert*-butyl)phenol [UV-327-6-*m*cx; chemical purity > 98%], 2-(5-chloro-benzotriazol-2-yl)-4,6-di-(1-hydroxy-2-methylpropyl)phenol [UV-327-4 + 6-*di*OH; chemical purity 97%], 2-(5-chloro-benzotriazol-2-yl)-4-(1-carboxy-2-methylpropyl)-6-(1-hydroxy-2-methylpropyl)phenol [UV-327-4-*m*cx-6-*m*OH; chemical purity > 97%], and 2-(5-chloro-benzotriazol-2-yl)-6-(1-carboxy-2-methylpropyl)-4-(1-hydroxy-2-methylpropyl)phenol [UV-327-4-*m*OH-6-*m*cx; chemical purity > 97%]. The internal standards 2-(5-chloro-benzotriazol-2-yl)-4-(1,1-di-([^2^H_3_]-methyl)-2-hydroxyethyl)-6-(*tert*-butyl)phenol [D_6_-UV-327-4-*m*OH; chemical purity 99%; isotopic purity > 98%], 2-(5-chloro-benzotriazol-2-yl)-4-(1,1-di-([^2^H_3_]-methyl)-1-carboxymethyl)-6-(*tert*-butyl)phenol [D_6_-UV-327-4-*m*cx; chemical purity 98%; isotopic purity > 98%], 2-(5-chloro-benzotriazol-2-yl)-6-(1,1-dimethyl-1-carboxymethyl)-4-(1,1-di-([^2^H_3_]-methyl)-2-hydroxyethyl)phenol [D_6_-UV-327-4-*m*OH-6-*m*cx; chemical purity 95%; isotopic purity > 98%], and 2-(5-chloro-benzotriazol-2-yl)-4,6-di-[1,1-di-([^2^H_3_]-methyl)-2-hydroxyethyl]phenol [D_12_-UV-327-4 + 6-*di*OH; chemical purity 96%; isotopic purity > 98%] were also synthesized by the Institute for Organic and Biomolecular Chemistry (Göttingen, Germany). Acetonitrile [anhydrous] was purchased from VWR International GmbH (Darmstadt, Germany). Acetone [GC grade], ammonium acetate, chloroform [GC grade], ethyl acetate [p.a.], glacial acetic acid, hydrochloric acid [HCl], isopropanol [GC grade], *n*-hexane [GC grade], *N,O*-bis(trimethylsilyl)acetamide in combination with 5% trimethylchlorosilane [BSA/TMCS], *N*-(trimethylsilyl)imidazole [TSIM], sodium chloride [NaCl], sodium hydroxide [NaOH], and toluene [GC grade] were ordered from Merck KGaA (Darmstadt, Germany). *β*-Glucuronidase/arylsulfatase from *Helix pomatia* (*H. pomatia*) was purchased from Roche Diagnostics GmbH (Mannheim, Germany). Double distilled water was prepared using a Milli-Q system (Millipore, Bedford, USA). Human plasma was obtained from in.vent Diagnostica GmbH (Hennigsdorf, Germany). Human blood was donated by a volunteer participating in the *in* *vivo* metabolism study.

### Study design

Three healthy volunteers (two men and one woman) aged between 22 and 56 years were included in the study. Table 1 summarizes further information on the study participants. The administered dose of UV-327 was calculated with respect to an adequate distance from the no observed adverse effect level (NOAEL) determined in toxicological studies. Thus, an NOAEL of 30 mg/kg/day, which was established in a 90-day feeding study with beagle dogs (CIBA AG 1970), was used as a point of departure and combined with a safety factor of 100. As a result, 0.3 mg of UV-327/kg body weight was administered to each participant.

**Table 1:** Information on the participants of the in vivo study with single oral administration of UV-327 (0.3 mg/kg body weight)

Subject	Gender	Age [years]	Body weight [kg]	Number of urine samples	Total volume of urine [l]	Number of blood samples	Number of plasma samples
1	Male	56	88	43	4.9	13	13
2	Male	27	97	33	4.7	10	–
3	Female	22	45	30	3.1	10	–

For the determination of potential background exposure, one blood and one urine sample of each participant were collected prior to the oral administration of UV-327. 14–29 mg of UV-327 were weighed directly onto a small piece of bread with butter, which was then consumed by the volunteers. Samples of urine and blood were then collected up to 72 h post-application. The participants collected all urine voids in separate containers. The sampling times and volumes of each sample were recorded. The urine samples were aliquoted and stored at − 50 °C until analysis. 9 ml of blood were drawn from peripheral veins at 2 h, 4 h, 6 h, 8 h, 10 h, 24 h, 34 h, 48 h, and 72 h after exposure and collected in EDTA-Monovettes^®^. Additional blood samples were collected from one subject 14 h, 28 h, and 58 h after exposure. This study participant donated two blood samples at each point in time; one blood sample was centrifuged to obtain plasma samples. Both blood and plasma samples were stored at − 50 °C until analysis.

The ethics committee of the Friedrich-Alexander-Universität Erlangen-Nürnberg approved the study design (49_18 B). All participants were informed about the goals and risks of the study and gave written, informed consent. General inclusion criteria for study participation were an age of between 18 and 60 years, nonsmoker status, and an absence of occupational exposure to UV-327. All participants met these criteria.

### Sample preparation

#### Urine samples

The urine samples were prepared, processed, and analyzed according to a previously published method (Fischer and Göen 2021). Enzymatic hydrolysis was performed at 37 °C for 16 h using *β*-glucuronidase/arylsulfatase from *H.* *pomatia*. The analytes and internal standards were extracted by dispersive liquid–liquid microextraction (DLLME) with isopropanol and chloroform. The resulting extracts were evaporated to dryness, followed by the addition of toluene and derivatization with both BSA/TCMS and TSIM. The limits of detection (LODs) ranged from 0.05 to 0.10 µg/l. Precision and repeatability were confirmed by relative standard deviations below 15%. Mean relative recovery rates ranged from 88% to 112%. The urinary creatinine content was determined photometrically by the Jaffé’s method (Larsen 1972).

The urine samples of one study participant were additionally processed without the addition of *β*-glucuronidase/arylsulfatase from *H.* *pomatia* or incubation at 37 °C to quantify the unconjugated forms of UV-327 and its metabolites in urine.

#### Blood samples

Whole blood samples were prepared, processed, and analyzed according to a previously published method (Fischer and Göen 2022). Proteins and cellular components were precipitated by the addition of acetonitrile. After centrifugation, the supernatants were diluted with water and ammonium acetate buffer. For analyte extraction, chloroform was added. The extracts were evaporated to dryness, followed by the addition of toluene and derivatization with both BSA/TCMS and TSIM. LODs ranged from 0.02 to 0.36 µg/l. Precision and repeatability were confirmed by relative standard deviations below 15%. Mean relative recovery rates ranged from 91% to 118%.

The blood samples of one study participant were additionally processed with the addition of *β*-glucuronidase/arylsulfatase from *H.* *pomatia* and incubation at 37 °C for 16 h to examine potential conjugation of UV-327 and its metabolites to glucuronide/sulfate.

#### Plasma samples

Stock solutions of each analyte and internal standard (200 mg/l) were prepared in acetone. For calibration, an analyte spiking solution was prepared in a mixture of acetonitrile and aqueous 0.9% NaCl solution (v/v, 1:1), which contained 500 µg/l of each metabolite. For the determination of UV-327, two analyte spiking solutions were prepared, containing 2 mg/l or 40 mg/l of UV-327 in a mixture of acetonitrile and 0.9% NaCl solution (v/v, 1:1). The internal standard spiking solution contained 20 mg/l of D_20_-UV-327 and 1 mg/l each of D_6_-UV-327-4-*m*cx, D_6_-UV-327-4-*m*OH, D_12_-UV-327-4 + 6-*di*OH, and D_6_-UV-327-4-*m*OH-6-*m*cx. For derivatization, a solution containing 5% (v/v) TSIM in toluene was freshly prepared for each analytical run. Calibration standards were prepared by spiking human plasma with various volumes of the analyte spiking solutions. An eight-point calibration curve in the range of 0.5–25 µg/l was applied for the determination of the metabolites, except for UV-327-4-*m*OH-6-*m*cx, for which the calibration range was set to 2–100 µg/l due to higher limits of detection and quantitation. For the determination of UV-327, a nine-point calibration curve in the range of 10–2000 µg/l was applied. A reagent blank sample, containing water instead of plasma, was included in each analytical run. Any blank values were subtracted from the analytical results. One sample each of *Q*_low_ (low-concentration quality-control material), *Q*_mid_ (medium-concentration quality-control material), and *Q*_high_ (high-concentration quality-control material) was processed analogously to the samples in each analytical run. The concentrations of the quality-control materials are given in Table SI-1.

Plasma samples were, with some minor modifications, processed according to a method published by Andrenyak et al. (2017). The samples were thawed at room temperature and thoroughly mixed on a roller mixer. Subsequently, 1 ml of plasma was pipetted into an 8-ml glass vial. Both 10 µl of the internal standard spiking solution and 2 ml of acetonitrile were added to the samples, which were immediately vortex-mixed for 1 min. After centrifugation at 1400×*g* for 10 min, the supernatants were transferred into clean 8-ml glass vials and the volume was reduced to ≈ 1 ml under a stream of nitrogen. Both 1 ml of 1 M HCl and 3 ml of a mixture of hexane and ethyl acetate (9:1) were then added to the samples. The samples were mixed on a laboratory shaker for 20 min. After centrifugation at 1400×*g* for 10 min, the upper organic phases were transferred into clean 8-ml glass vials and the volume of each sample was reduced to ≈ 1 ml under a stream of nitrogen. The samples were then transferred into 2-ml glass vials and evaporated to dryness. Following the addition of 40 µl of toluene, the samples were derivatized with 40 µl of BSA/TMCS and 20 µl of 5% (v/v) TSIM in toluene.

The supplemental material contains a description of the validation procedure. LODs ranged from 0.03 to 0.86 µg/l with corresponding limits of quantitation (LOQs) from 0.11 to 2.9 µg/l. The detailed validation data are given in Table SI-2.

### Instrumentation

A TRACE 1310 gas-chromatographic system equipped with a TriPlus RSH autosampler and a split/splitless injector was used for the analysis of the urine, blood, and plasma samples. The gas-chromatographic system was coupled to a TSQ 9000 triple–quadrupole mass spectrometer equipped with an advanced electron ionization (AEI) source (Thermo Fisher Scientific Inc., Waltham, USA). Chromatographic separation was performed on a (5% phenyl)-methylpolysiloxane low-bleed capillary column (HP-5msUI, 60 m × 250 µm × 0.25 µm, Agilent Technologies, Inc., Santa Clara, USA) at a constant flow rate of 1 ml/min using helium as a carrier gas. Total analysis time was 44 min. The GC–MS/MS equipment and parameter-specific settings were used as described in earlier publications for the analysis of urine (Fischer and Göen 2021) and blood samples (Fischer and Göen 2022). Plasma samples were analyzed using the same parameter-specific settings as for the analysis of blood samples.

### Data evaluation

Following an exploratory approach, we included all detectable results in the toxicokinetic analysis to elucidate human in vivo metabolism and time courses as entirely as possible.

Renal excretion rates (*R*_E_, in µg/h) of each analyte at a certain point in time were calculated by the following equation:

$${R}_{\mathrm{E}}= \frac{{c}_{i} \times {v}_{i}}{{t}_{i} -{t}_{i-1}}$$

where *c*_*i*_ (in µg/l) is the concentration of UV-327 or its metabolites in the urine sample *i*, *v*_*i*_ (in l) is the volume of the respective urine sample, *t*_*i*_ (in h) is the elapsed time value of the sampling, and *t*_*i*−1_ (in h) is the elapsed time value of the previous sampling.

Renal excretion kinetics of UV-327 and its metabolites were plotted as temporal progressions of the renal excretion rates *R*_*E*_ at the midpoint of the respective sampling periods (*t*_*i*,m_ in h), which were calculated as follows:

$$t_{{i,{\text{m}}}} = t_{{i{-}1}} + \frac{{t_{i} - t_{{i{-}1}} }}{2}$$

Excretion curves were prepared for each study participant and each analyte by plotting the current excretion rates against the average time of the sampling period. Mean excretion curves were then obtained by averaging the closest sampling time points and the corresponding renal excretion rates for all study participants (mean ± standard deviation (SD)). The slopes (*k*_el_, elimination rate constant) of the ln-transformed mean excretion curves were used to calculate the elimination half-lives (*t*_1/2_) as follows:

$${t}_{1/2}=\frac{\mathrm{ln}(2)}{\left|{k}_{\mathrm{el}}\right|}$$

By summing the molar excreted amounts of UV-327 and its metabolites, the cumulative excreted amount of each analyte (in µmol) was calculated for each study participant:

$${\sum }_{i=0}^{n}\frac{{c}_{i}\times {v}_{i}}{M}$$

where *M* (in µg/µmol) is the molar mass of the respective analyte. Furthermore, urinary excretion factors (*F*_UE_) as UV-327 dose equivalents (as percentages) were calculated to express the total excretion of UV-327 and its metabolites in urine after 24 h, 48 h, and 72 h:

$${F}_{\mathrm{UE}}= \frac{{\mathrm{CE}}_{i}}{{M}_{\mathrm{D}}}\times 100$$

*CE*_*i*_ is the amount of the respective analyte excreted after 24 h, 48 h, and 72 h (in µmol) and *M*_D_ is the ingested amount of UV-327 (in µmol).

The shares of conjugation of UV-327 and its metabolites were determined by correlation of the urinary excretion rates obtained with and without the performance of enzymatic hydrolysis. The slope of the linear fit describes the percentage of the unconjugated form of the respective analyte.

Mean concentration–time curves in blood were obtained by plotting the mean blood levels against time. The elimination half-lives (*t*_1/2_) were calculated as described above. The maximum blood levels of all analytes were considered as the most suitable time points for approximation of an overall distribution function in blood. For this purpose, UV-327 equivalents (as percentages) were calculated as follows:

$$\sum_{i=0}^{n}\frac{{c}_{\mathrm{max}}}{M} \times {M}_{\mathrm{UV}-327} \times 100$$

where *c*_max_ is the maximum blood level (in µg/l), *M* (in µg/µmol) is the molar mass of the respective analyte, and *M*_UV-327_ (in µg/µmol) is the molar mass of UV-327. Plasma-to-blood ratios were obtained from the concentration–time curves of the study participant who donated both plasma and blood samples. The areas under the concentration–time curves of UV-327 and its metabolites in plasma were, therefore, divided by the areas under the concentration–time curves in blood. The areas under the curve were calculated using the trapezoidal method.

Microsoft Excel^®^ was used for data processing and Origin^®^ was used for curve fitting.

## Results

### UV-327 and its metabolites in blood

**Fig. 2:**
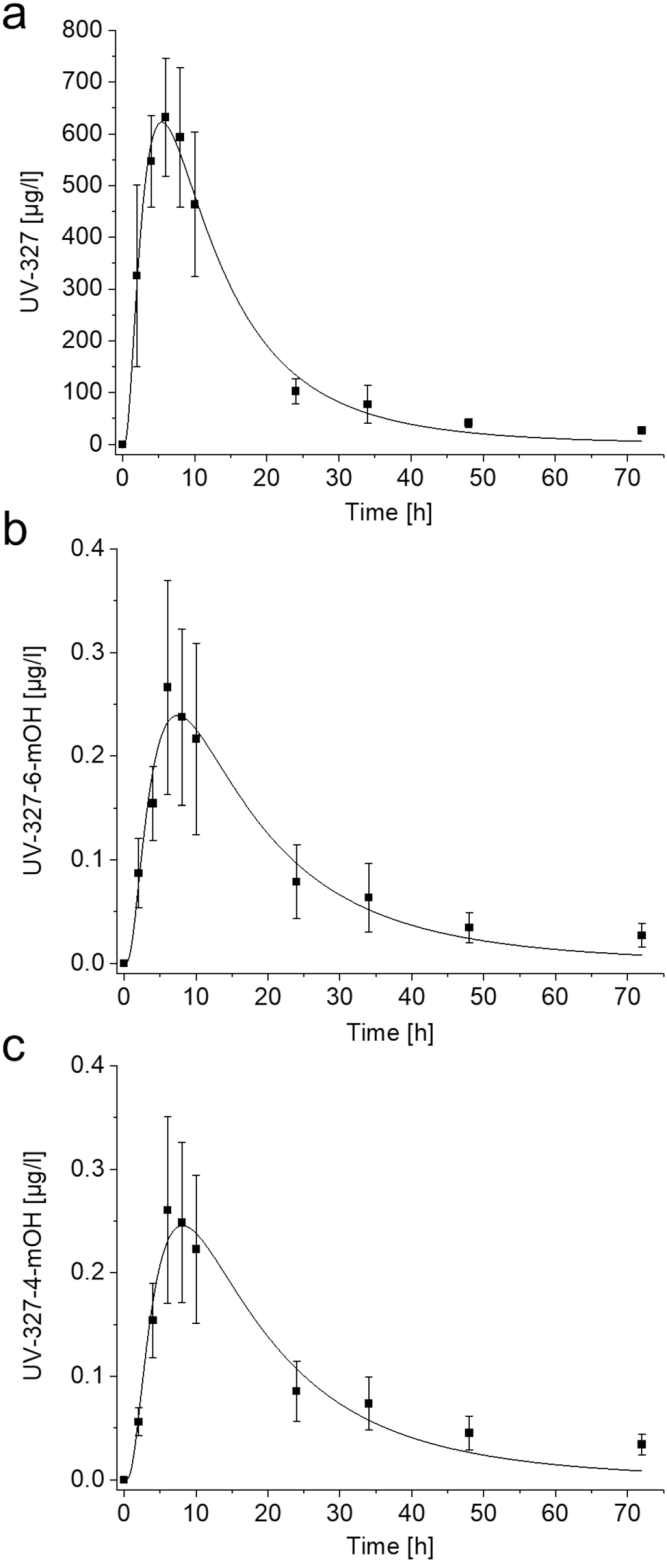
Mean concentrations of **a** UV-327, **b** UV-327-6-*m*OH, and **c** UV-327-4-*m*OH in blood after single oral administration of UV-327 with log-normal fit (*n* = 3, mean ± SD)

In the blood samples collected before oral administration of UV-327, none of the metabolites were detected. UV-327 was detected in these samples, but only at marginal levels and mostly below the LOD. UV-327, UV-327-4-*m*OH, and UV-327-6-*m*OH were detected in the blood samples collected after exposure to UV-327. The toxicokinetic data of UV-327 and its metabolites in blood are summarized in Table 2. Figure 2 shows the concentration–time curves of UV-327, UV-327-6-*m*OH, and UV-327-4-*m*OH in blood. The mean maximum blood level (*c*_max_ ± SD) of UV-327 (632 ± 114 µg/l) was reached 6 h after oral administration. Afterward, an initial moderate decline in the concentration was observed. Twenty-four hour post-exposure, the mean blood level of UV-327 was 103 ± 24 µg/l, followed by a slower terminal elimination phase. At 72 h post-exposure, 27 ± 5 µg/l of UV-327 were still detectable, which demonstrates that the elimination of the parent compound was not complete 3 days after exposure. Mean maximum blood levels of the two monohydroxylated metabolites UV-327-6-*m*OH (0.27 ± 0.10 µg/l) and UV-327-4-*m*OH (0.26 ± 0.10 µg/l) were also reached 6 h after oral administration of UV-327. Afterward, an initial moderate decline of their levels was observed as well. Twenty-four hour post-exposure, the mean blood levels of UV-327-6-*m*OH and UV-327-4-*m*OH were 0.08 ± 0.04 µg/l and 0.09 ± 0.03 µg/l, respectively. Thereafter, a slower decline in the mean concentrations was observed until the mean blood levels of both metabolites were below their respective LODs after 48 h (UV-327-6-*m*OH) and 72 h (UV-327-4-*m*OH). Elimination half-lives (t_1/2_) of UV-327, UV-327-6-*m*OH, and UV-327-4-*m*OH for phases 1 and 2 were estimated to be 6.6–10.8 h and 24.9–33.0 h, respectively. No further metabolites were detected in any of the collected blood samples.

**Table 2:** Kinetics of UV-327, UV-327-4-*m*OH, and UV-327-6-*m*OH in blood after single oral administration of UV-327 (*n* = 3; mean ± SD)

	UV-327	UV-327-6-*m*OH	UV-327-4-*m*OH
*c*_max_ [µg/l]	632 ± 114	0.27 ± 0.10	0.26 ± 0.10
*t*_max_ [h]	6	6	6
*t*_1/2_—phase 1 [h]	6.6 ± 0.3	9.9 ± 1.0	10.8 ± 0.3
*t*_1/2_—phase 2 [h]	24.9 ± 1.9	29.0 ± 1.1	33.0 ± 0.2

*C*_*max*_ maximum blood level, *t*_*max*_ time point of maximum blood level, *t*_*1/2*_ elimination half-life

UV-327 accounted for 99.9% of the total amount of all analytes at the time point of maximum concentration, while UV-327-6-*m*OH and UV-327-4-*m*OH each accounted for only 0.04%. The analyte concentrations obtained with enzymatic hydrolysis were comparable with the concentrations obtained without enzymatic hydrolysis. UV-327 and its monohydroxylated metabolites are, therefore, not present as conjugates in blood.

### UV-327 and its metabolites in plasma

**Fig. 3:**
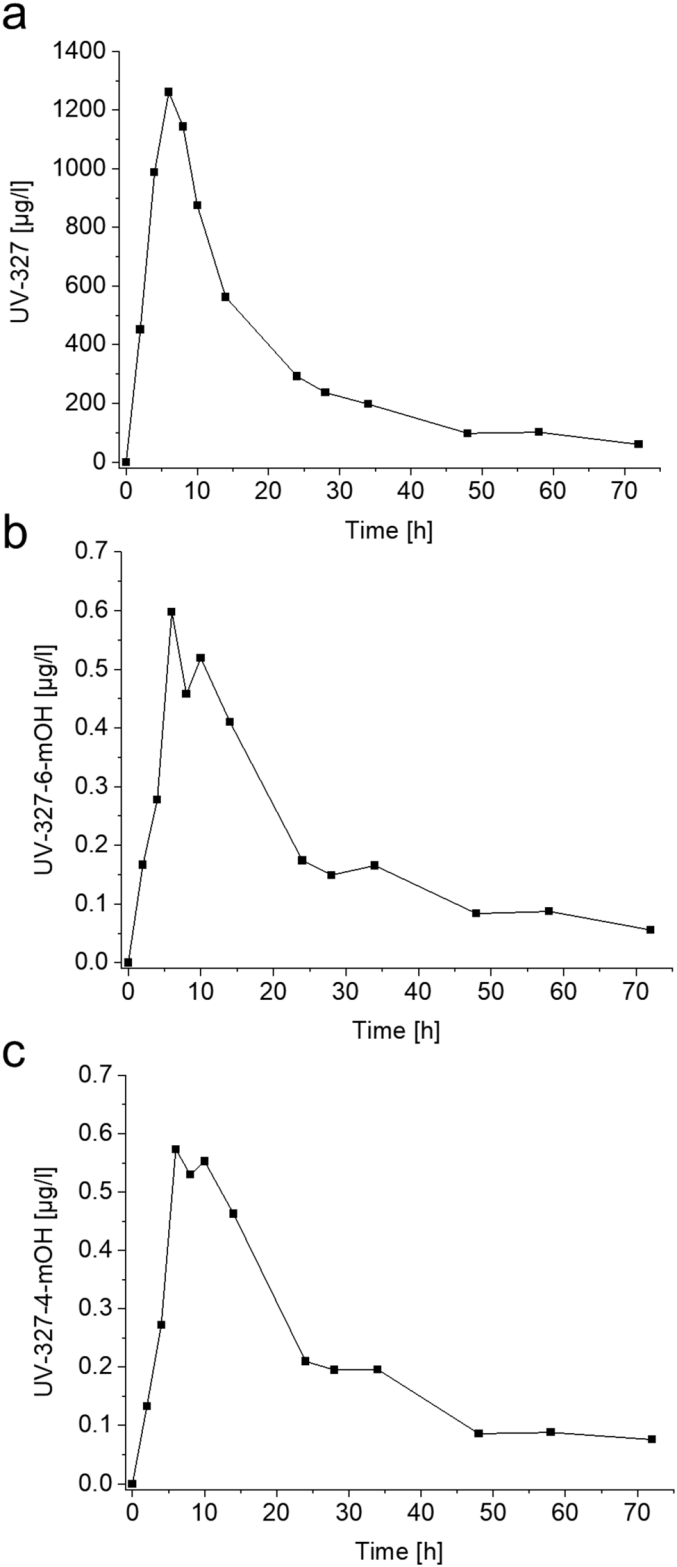
Plasma concentration–time curves of **a** UV-327, **b** UV-327-6-*m*OH, and **c** UV-327-4-*m*OH (*n* = 1)

**Fig. 4:**
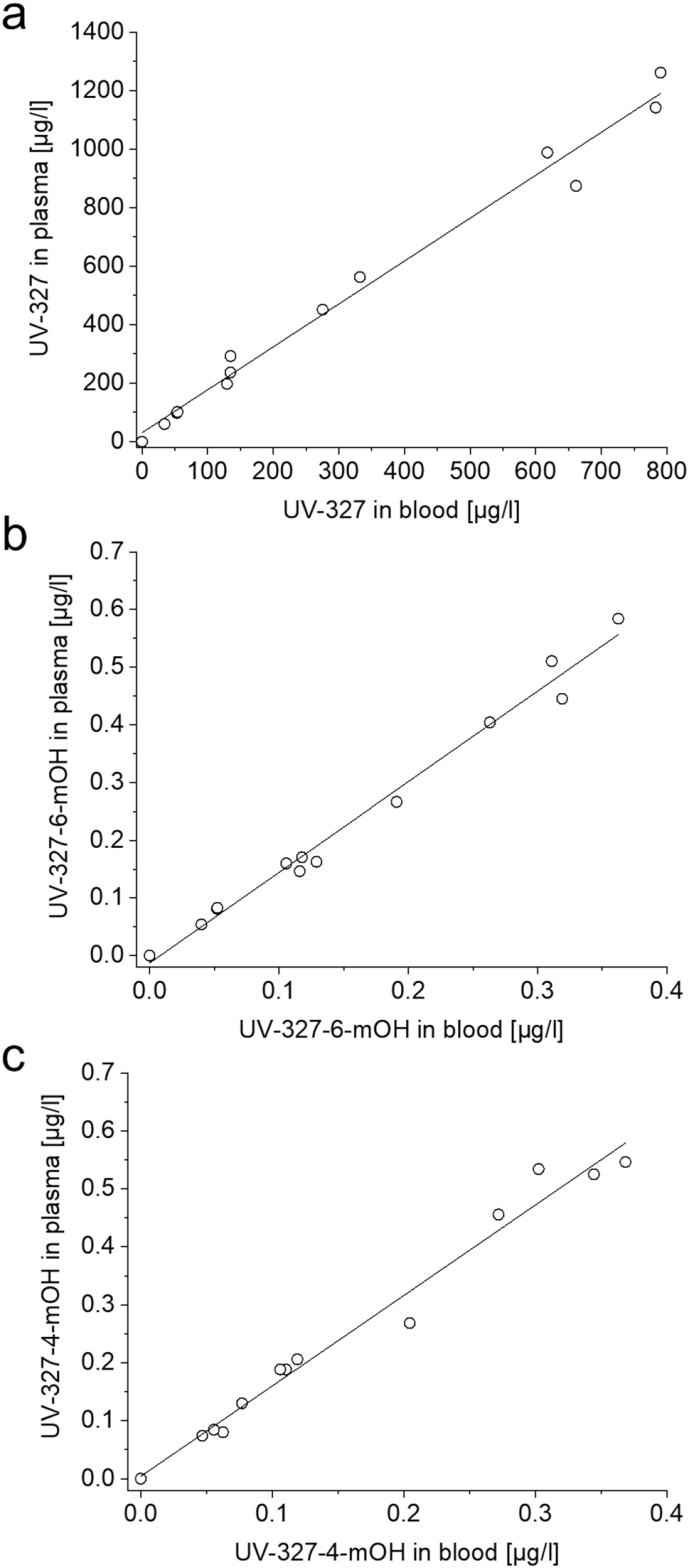
Correlations between **a** UV-327, **b** UV-327-6-*m*OH, and **c** UV-327-4-*m*OH concentrations in plasma and blood samples (*n* = 1)

In the plasma sample collected before oral administration of UV-327, none of the metabolites were detected. The concentration of UV-327 in the sample collected before exposure was equal to the LOD. Figure 3 shows the plasma concentration–time curves of UV-327, UV-327-6-*m*OH, and UV-327-4-*m*OH. Maximum plasma levels of UV-327 (1261 µg/l), UV-327-6-*m*OH (0.58 µg/l), and UV-327-4-*m*OH (0.55 µg/l) were reached 6 h post-exposure. Linear correlations were observed between the plasma and blood levels of UV-327 (*R*^2^ = 0.9856, *y* = 1.4668*x* + 30.674), UV-327-6-*m*OH (*R*^2^ = 0.9857, *y* = 1.5706*x* − 0.0126), and UV-327-4-*m*OH (*R*^2^ = 0.9787, *y* = 1.5605*x* + 0.0041) (Fig. 4).

### UV-327 and its metabolites in urine

In the urine samples collected before oral administration of UV-327, none of the analytes were detected (< LOD). In the urine samples collected after oral administration of UV-327, four analytes were detected, namely, UV-327, UV-327-4-*m*OH, UV-327-6-*m*OH, and UV-327-4 + 6-*di*OH. The renal excretion kinetics of UV-327 and its metabolites are summarized in Table 3. Maximum urinary excretion rates were reached after 9–14 h. For UV-327, mean maximum urinary excretion rates of 0.026 ± 0.007 µg/h were reached 13.5 ± 0.05 h post-exposure. Mean maximum urinary excretion rates of UV-327-6-*m*OH (0.068 ± 0.06 µg/h) and UV-327-4-*m*OH (0.032 ± 0.012 µg/h) were each reached 8.7 ± 0.21 h and 13.5 ± 0.05 h after oral administration of UV-327, respectively. The dihydroxylated and thus most polar metabolite UV-327-4 + 6-*di*OH exhibited the highest mean maximum excretion rates (0.214 ± 0.058 µg/h), which were reached 10.6 ± 0.08 h post-exposure.

**Table 3:** Renal excretion kinetics of UV-327, UV-327-4-*m*OH, UV-327-6-*m*OH, and UV-327-4 + 6-*di*OH after single oral administration of UV-327 (*n* = 3; mean ± SD)

	UV-327	UV-327-6-*m*OH	UV-327-4-*m*OH	UV-327-4 + 6-*di*OH
RE_max_ [µg/h]	0.026 ± 0.007	0.068 ± 0.06	0.032 ± 0.012	0.214 ± 0.058
*t*_max_ [h]	13.5 ± 0.05	8.7 ± 0.21	13.5 ± 0.05	10.6 ± 0.08
*t*_1/2_ [h]	18.2	13.9	26.7	33.0
Cumulative excreted amount [µmol]	0.0016 ± 0.0007	0.0030 ± 0.0011	0.0016 ± 0.0009	0.0121 ± 0.0051
F_UE_ after 24 h [%]	0.0015 ± 0.0004	0.0034 ± 0.0006	0.0016 ± 0.0004	0.0104 ± 0.0022
F_UE_ after 48 h [%]	0.0023 ± 0.0007	0.0046 ± 0.0010	0.0022 ± 0.0007	0.0156 ± 0.0027
F_UE_ after 72 h [%]	0.0026 ± 0.0009	0.0049 ± 0.0013	0.0026 ± 0.0011	0.0183 ± 0.0030
Share of conjugation [%]	16	100	100	100

*RE*_*max*_ maximum renal excretion rate, *t*_*max*_ time point of maximum renal excretion rate, *t*_*1/2*_ elimination half-life, *F*_*UE*_ urinary excretion factor

**Fig. 5:**
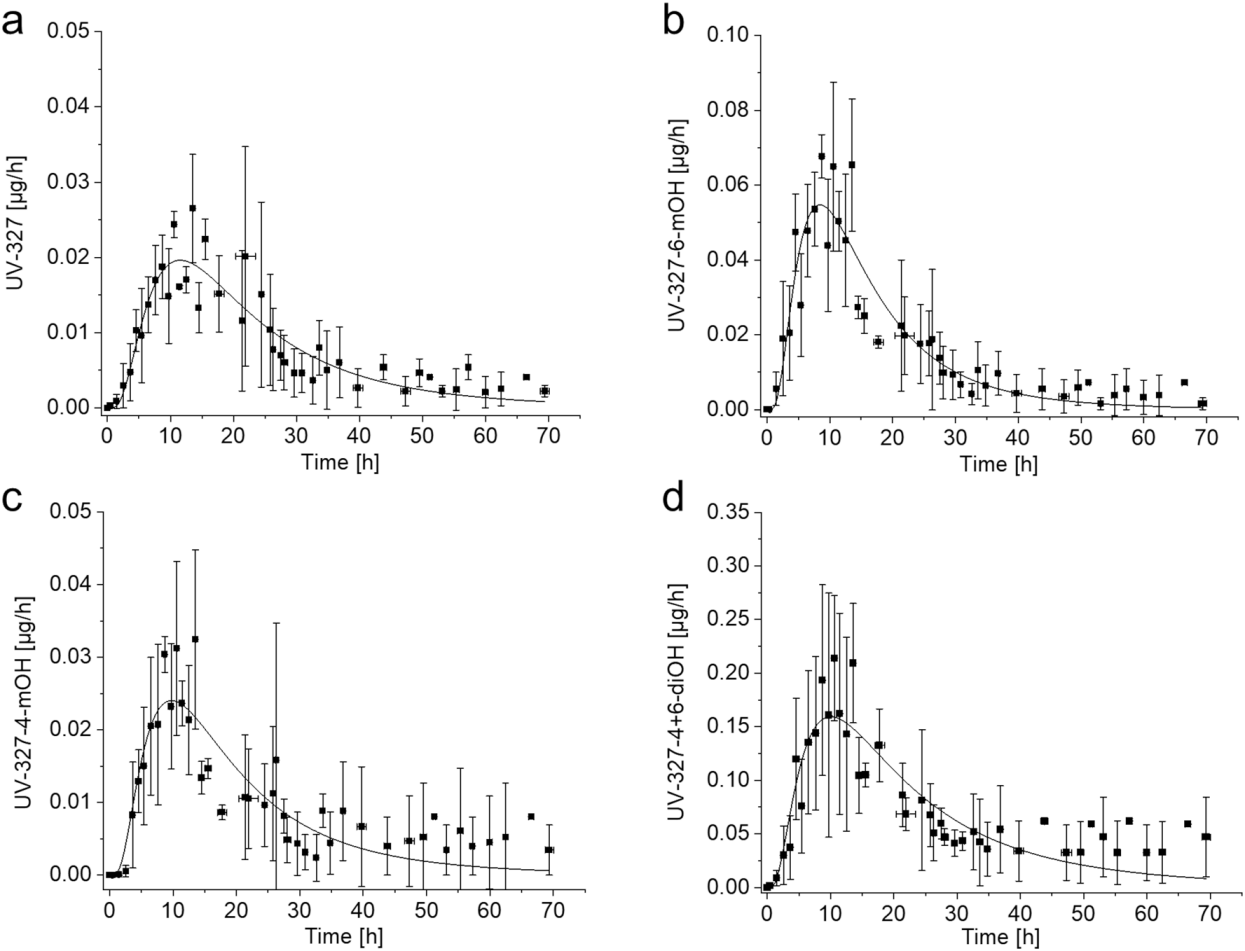
Mean urinary excretion rates of **a** UV-327, **b** UV-327-6-*m*OH, **c** UV-327-4-*m*OH, and **d** UV-327-4 + 6-*di*OH after single oral administration of UV-327 with log-normal fit (*n* = 3, mean ± SD)

Figure 5 shows the temporal progressions of the urinary excretion of UV-327 and its mono- and dihydroxylated metabolites. Due to the very low urinary excretion rates of UV-327 and its hydroxylated metabolites, as well as interindividual differences between the study participants, only one mean elimination half-life could be reliably determined for each analyte, as a clear differentiation of elimination phases was not possible.

The overall urinary excretion rates of UV-327 and its metabolites were very low. Within 72 h, only 0.03% of the orally administered dose of UV-327 was recovered in urine as UV-327 and its metabolites. Among the analytes found in urine, UV-327-4 + 6-*di*OH was the main metabolite and accounted for 0.018% of the applied dose; it accounted for 64.5% of the dose that was recovered in urine within 72 h. Within 24 h and 48 h, a respective 61% and 88% of the total urinary amount, recovered as UV-327 and its metabolites within 72 h, have been excreted.

Processing the urine samples with and without enzymatic hydrolysis revealed that UV-327 is mainly present in its unconjugated form (84%), whereas UV-327-6-*m*OH, UV-327-4-*m*OH, and UV-327-4 + 6-*di*OH are only present in their conjugated forms (see Table 3).

## Discussion

The relatively high peak level of the parent compound in blood indicates a quantitatively high oral absorption rate. However, maximum blood levels of UV-327 were reached only after 6 h, which is much later than after oral administration of other chemicals and, therefore, may indicate a temporally retarded absorption of UV-327 through the intestinal mucosa. For example, propyl paraben reached maximum serum levels 1.4 ± 1.1 h after oral administration (Shin et al. 2019) and the natural product *N,N*-dimethyltyramine reached plasma peak levels 30–90 min after oral administration (Sommer et al. 2020). Nevertheless, the structurally related BUVS 2-(2*H*-benzotriazol-2-yl)-4,6-di-*tert*-pentylphenol (UV-328) showed retarded resorption kinetics as well and reached maximum blood levels 8 h after oral administration (Denghel et al. 2021). The comparable findings for UV-327 and UV-328 indicate similar resorption processes for these substituted BUVSs.

In spite of an extensive monitoring of probable metabolites, only few of them as well as very low levels of them were found in blood/plasma and urine. Accordingly, UV-327 was only moderately metabolized in in vitro experiments with human liver microsomes (Fischer et al. 2020). Moreover, lower metabolism in substituted BUVSs compared to unsubstituted BUVSs was observed in an animal study (Waidyanatha et al. 2021). The low rate of metabolism of UV-327 might be related to the low reactivity of its bulky *tert*-butyl substituents (OECD 2017).

Due to their lipophilic properties (Log *K*_ow_ (UV-327) = 6.75 (Cantwell et al. 2015)), UV-327 and its hydroxylated metabolites might be reabsorbed from the intestine with subsequent enterohepatic circulation, which prolongs their residence time in the body and leads to an extended elimination half-life. In fact, the mean terminal elimination half-lives of UV-327 and its monohydroxylated metabolites in blood ranged from 24.9 to 33.0 h, whereby the elimination half-lives of the metabolites are somewhat longer than the elimination half-life of the parent compound. UV-327 was furthermore still detectable in relatively high concentrations 72 h post-exposure, indicating that its elimination from the body takes at least several days. Due to the distinctive hydrophobicity, storage in adipose tissue as well as in other tissues and organs is conceivable, which would also lead to a prolonged elimination half-life. Consistent with this conclusion, UV-327 has already been detected in adipose tissue (Yanagimoto et al. 2011) as well as breast milk (Kim et al. 2019; Lee et al. 2015; Sun et al. 2022).

Higher levels in plasma compared to whole blood were observed and can be attributed to the volume displacement by cellular components of whole blood (Ehresman et al. 2007). The higher plasma compared to whole blood levels result from the lower total sample volume after removing the cellular components by centrifugation. As a result, the analytes are enriched in the plasma fraction (González-Domínguez et al. 2020). The plasma to blood ratios of UV-327, UV-327-6-*m*OH, and UV-327-4-*m*OH were 1.47, 1.57, and 1.56, respectively, indicating that the binding and resorption of UV-327 and its monohydroxylated metabolites to erythrocytes is negligible.

Urinary excretion of UV-327 and its metabolites occurs slowly and only to a small extent. Only hydroxylated metabolites were detected, whereas higher oxidized metabolites (carboxylated metabolites and hydroxylated as well as carboxylated metabolites), which were found in in vitro experiments with human liver microsomes (Fischer et al. 2020), were detected neither in blood/plasma nor in urine. The *in vivo* biotransformation pathway of UV-327, therefore, appears to be less extensive than its in vitro metabolism pathway (see Fig. 1). The reason for the absence of higher oxidized products in blood/plasma and urine may be the rapid elimination of the hydroxylated products via urine immediately after generation. They may rapidly undergo phase II reactions leading to elimination before higher oxidized products are formed. This hypothesis is supported by the fact that each of the hydroxylated metabolites were completely conjugated when excreted with the urine. In contrast, the parent compound was excreted mainly without conjugation, which indicates the low accessibility of the phenolic hydroxyl group for phase II enzymes, whereas the additional hydroxyl groups of the UV-327 metabolites are easily accessible for the conjugation of glucuronic acid or sulfate.

The relative urinary shares of UV-327-4 + 6-*di*OH, UV-327-6-*m*OH, UV-327-4-*m*OH, and UV-327 were 64.5%, 17.2%, 9.2%, and 9.0% of the amount recovered within 72 h, respectively. Thus, the relative proportions of the analytes in blood and urine differed considerably. In blood, mainly UV-327 was found (99.9% of the total amount of analytes found in blood at the time point of maximum concentration), whereas UV-327 accounted for only 9.0% of the amount recovered in urine. UV-327-4 + 6-*di*OH was not detected in blood, but was the main metabolite in urine. The shift of the metabolites’ shares toward the dihydroxylated metabolite may be explained by its higher polarity, which favors renal excretion.

Since UV-327 and its metabolites in urine accounted for only about 0.03% of the orally administered dose of UV-327, it is theoretically conceivable that further metabolites are formed which were not identified in the in vitro experiments and are, therefore, not detectable by available analytical methods. Due to their hydrophobicity and relatively high molecular weights, the more likely reason for the low urinary levels of UV-327 and its metabolites is, however, that they may be excreted predominantly with the bile. Prolonged elimination half-lives are expected, since substances excreted via the biliary tract are subject to enterohepatic circulation. Accordingly, mean elimination half-lives of 18.2 h (UV-327), 13.9 h (UV-327-6-*m*OH), 26.7 h (UV-327-4-*m*OH), and 33.0 h (UV-327-4 + 6-*di*OH) were observed. Another indication for bile being the main route of elimination is that UV-327 is well-absorbed from the intestine, as is reflected in its high blood levels, but its urinary excretion rates are very low. Most of the substance must, therefore, be excreted via an alternative route. Consistently, biliary excretion was assumed to be the main route of excretion for UV-328, too (Denghel et al. 2021).

## Conclusion

The *in vivo* study presented herein provides initial information on the absorption, metabolism, and elimination of the UV absorber UV-327. The data indicate that UV-327 is quantitatively well-absorbed from the intestine, but is eliminated from the body with relatively slow kinetics. Concurrently, only minor amounts of the substance are metabolized. In total, three metabolites of UV-327, which carry hydroxyl groups at one or both *tert*-butyl groups, were detected in the studied samples. In blood and urine, the monohydroxylated metabolites UV-327-4-*m*OH and UV-327-6-*m*OH were found. In urine, the dihydroxylated metabolite UV-327-4 + 6-*di*OH was additionally detected and was the main metabolite accounting for 64.5% of the dose recovered within 72 h. Carboxylated metabolites and hydroxylated as well as carboxylated metabolites, which have been confirmed to be formed *in* *vitro*, could not be detected in any of the *in* *vivo* samples. UV-327 and its biotransformation products seem to be mainly eliminated via the feces, while urinary excretion is only a minor route of elimination with only about 0.03% of the administered dose being detected in urine samples up to 72 h post-exposure. Due to their lipophilic properties, the minor relevance of renal elimination, and the slow elimination kinetics, UV-327 and its metabolites may accumulate in the human body with repeated exposure. The present study complements the insight in the complex absorption, distribution, metabolism, and elimination (ADME) processes of BUVSs.

## Supplementary Information

Below is the link to the electronic supplementary material.

Supplementary file1 (PDF 570 KB)
