# Supplementary material for: Human metabolism and excretion kinetics of benzotriazole UV stabilizer UV-327 after single oral administration
Source: Arch Toxicol. 2022 Nov 5;97(1):165–76. doi: 10.1007/s00204-022-03401-3 (PMC9816242; doi:10.1007/s00204-022-03401-3)
Supplement: Supplementary file 1 — Supplementary file1 (PDF 570 KB) [file 204_2022_3401_MOESM1_ESM.pdf]

Supplementary Information

Human metabolism and excretion kinetics of benzotriazole UV stabilizer UV-327 after single oral administration

Corinna Fischer[1], Edgar Leibold[2], Julia Hiller[1], Thomas Göen[1,*]

[1]Institute and Outpatient Clinic of Occupational, Social and Environmental Medicine, Friedrich-Alexander-Universität Erlangen-Nürnberg, Henkestraße 9–11, 91054 Erlangen, Germany

[2]BASF SE, Product Safety, Carl-Bosch-Straße 38, 67056 Ludwigshafen am Rhein, Germany

[*]**Corresponding author:** thomas.goeen@fau.de

Table SI-1 Concentrations of the quality-control materials for the determination of UV-327 and its metabolites in plasma

Analyte	Q_{low} [µg/l]	Q_{mid} [µg/l]	Q_{high} [µg/l]
UV-327	20	200	800
UV-327-6-*m*cx	1	5	15
UV-327-6-*m*OH	1	5	15
UV-327-4-*m*OH	1	5	15
UV-327-4-*m*cx	1	5	15
UV-327-6-lactone	1	5	15
UV-327-4-*m*OH-6-*m*cx	4	20	60
UV-327-4+6-*di*OH	1	5	15
UV-327-4-*m*cx-6-*m*OH	1	5	15
UV-327-4-*m*OH-6-lactone	1	5	15

Q_{low} = low-concentration quality-control material, Q_{mid} = medium-concentration quality-control material, Q_{high} = high-concentration quality-control material

Validation procedure – plasma

To determine the limits of detection and quantitation, equidistant ten-point calibration curves were prepared, processed, and analyzed in triplicate in conjunction with a blank value (unspiked plasma samples). The limits of detection and quantitation were then calculated based on the standard deviations of the calibration curves according to the calibration-curve procedure (Bader et al. 2010; DIN 2008). The concentrations of the equidistant calibration curves ranged from 0.2 to 2.0 µg/l for UV-327, UV-327-4 *m*OH, UV-327-6-*m*OH, UV-327-4-*m*cx, UV-327-6-*m*cx, UV-327-4+6-*di*OH, and UV-327-4-*m*cx-6-*m*OH and from 2 to 20 µg/l for UV-327-4-*m*OH-6-*m*cx. Accuracy was determined at three different concentrations (Q_{low}, Q_{mid}, Q_{high}) by calculation of the relative recovery rates of five samples at each concentration level. Precision and repeatability were determined by calculating intraday and interday relative standard deviations at three different concentrations (Q_{low}, Q_{mid}, Q_{high}). To determine precision, the samples were processed and analyzed five times in parallel. Repeatability was determined by processing and analyzing the samples on four different days.

Table SI-2 Limits of detection and quantitation, precision (n = 5), repeatability (n = 4), and accuracy (n = 5) for the determination of UV-327 and its metabolites in plasma

Analyte	LOD [µg/l]	LOQ [µg/l]	Precision [%]			Repeatability [%]			Accuracy [%]		
			Q_{low}	Q_{mid}	Q_{high}	Q_{low}	Q_{mid}	Q_{high}	Q_{low}	Q_{mid}	Q_{high}
UV-327	0.05	0.17	3.0	4.6	2.2	11.9	6.2	3.3	96 ± 3	103 ± 5	102 ± 2
UV-327-6-*m*cx	0.22	0.68	19.7	14.8	18.5	15.5	12.3	12.0	77 ± 15	104 ± 15	116 ± 22
UV-327-6-*m*OH	0.05	0.16	4.1	1.6	1.8	5.1	2.5	5.6	97 ± 4	97 ± 2	97 ± 2
UV-327-4-*m*OH	0.05	0.16	3.9	1.4	1.3	4.2	2.0	1.8	95 ± 4	96 ± 1	97 ± 1
UV-327-4-*m*cx	0.05	0.18	3.3	5.6	3.3	5.1	3.0	3.2	92 ± 3	93 ± 5	97 ± 3
UV-327-6-lactone	0.08	0.26	3.8	4.3	8.8	3.5	2.9	5.1	90 ± 3	87 ± 4	97 ± 9
UV-327-4-*m*OH-6-*m*cx	0.86	2.90	2.3	9.3	7.3	6.1	6.3	7.3	117 ± 3	92 ± 9	91 ± 7
UV-327-4+6-*di*OH	0.03	0.11	3.6	3.9	1.6	3.1	2.5	1.8	97 ± 3	96 ± 4	101 ± 2
UV-327-4-*m*cx-6-*m*OH	0.07	0.22	3.6	3.1	2.9	5.5	6.6	5.5	90 ± 3	89 ± 3	95 ± 3
UV-327-4-*m*OH-6-lactone	0.06	0.21	2.1	3.3	8.8	8.5	4.6	10.2	85 ± 2	88 ± 3	103 ± 9

LOD = Limit of detection, LOQ = limit of quantitation, Q_{low} = low-concentration quality-control material, Q_{mid} = medium-concentration quality-control material, Q_{high} = high-concentration quality control-material

References

Bader M, Barr D, Göen T, Schaller KH, Scherer G, Angerer J (2010) Reliability criteria for analytical methods. Biomonitoring Methods, 2010. In: Angerer J., Hartwig A. (eds) The MAK-Collection for Occupational Health and Safety. Part IV: Biomonitoring Methods, vol 12. Wiley-VCH, Weinheim, pp 55–101

DIN (Deutsches Institut für Normung, German Institute for Standardization) (2008) DIN 32645:2008-11: Chemical analysis - decision limit, detection limit and determination limit under repeatability conditions - terms, methods, evaluation
